# Prevalence of radiographic findings in chronic osteomyelitis

**DOI:** 10.1186/s12891-023-07121-2

**Published:** 2024-01-18

**Authors:** Felipe Francisco Tuon, Celso Junio Aguiar Mendonça, Wagner Gasperin, Willian Lassalvia Zotto, Bruna Maria Stofela Sarolli, Janice Alexandra da Costa Manuel, June Alisson Westarb Cruz, Jamil Faissal Soni

**Affiliations:** 1 Laboratory of Emerging Infectious Diseases, School of Medicine, Pontifícia Universidade Católica Do Paraná, Curitiba, PR 80215-901 Brazil; 2 Department of Orthopedics, Hospital de Clínicas da UFPR, Curitiba, PR 80060-900 Brazil; 3 Department of Radiology, Hospital de Clínicas da UFPR, Curitiba, PR 80060-900 Brazil; 4 Academia BAI, Av. Comandante Loy, Edíficio A - Academia BAI, Entrada A, R/C Morro Bento, Luanda, Angola

**Keywords:** Osteomyelitis, Radiographical aspects, Infection, Bone, Sclerosis, Sequestrum

## Abstract

**Background:**

Simple radiography in conjunction with pertinent medical history and a comprehensive physical examination is typically adequate for diagnosing chronic osteomyelitis (CO). However, radiographic manifestations of CO lack specificity; therefore, the concordance among specialists in this regard has not been systematically assessed. This study aimed to compare and evaluate the proficiency of orthopedic surgeons and radiologists in identifying radiographic indicators present in simple radiographs for diagnosing CO.

**Methods:**

This cross-sectional study was a correlational investigation utilizing plain radiographs obtained from a cohort of 60 patients diagnosed with CO. Comprehensive assessments of the demographic and clinical characteristics, comorbidities, and microbiological parameters were conducted. Additional variables included the anatomical location of the CO, existence of fistulas, disease duration, and presence of pseudoarthrosis. This study meticulously documented the presence or absence of six specific findings: bone destruction, which incorporates erosion and radiolucencies around implants; bone sclerosis; cortical thinning concomitant with erosion; cortical thickening; sequestrum formation; and soft-tissue swelling.

**Results:**

Most patients were men (75%), with a mean age of 45.1 years. Hematogenous etiology of CO represented 23%. Bone sclerosis (71.3%) and cortical thickening (67.7%) were the most common radiographic findings, followed by soft-tissue swelling (51.3%), sequestration (47.3%), bone destruction (33.3%), and cortical erosion (30.3%). The mean agreement was 74.2%, showing a marked disagreement rate of 25.8% among all radiographic findings. The presence or absence of soft tissue edema, a prominent radiographic finding that was more important than the other findings, showed the greatest disagreement.

**Conclusions:**

Radiographic findings in CO were universally observed in all patients, demonstrating a high degree of concordance among specialists, with the exception of soft tissue swelling.

## Introduction

The prevalence of osteomyelitis has been increasing, primarily attributed to a surge in high-energy trauma, notably road traffic accidents, and an increased frequency of orthopedic surgical interventions [1]. Accurate diagnosis and timely intervention are imperative for the effective management of osteomyelitis. Notably, the estimated incidence of infections linked to orthopedic implants in the context of fracture fixation exceeds 100,000 cases annually, resulting in substantial impairment in physical function and a corresponding reduction in the overall quality of life [2, 3]. The incidence of infection in closed fractures is generally low, typically ranging between 1 and 2%. In contrast, open fractures exhibit a markedly higher susceptibility to infection, with up to 30% of cases evolving into infectious complications [4–7]. Inadequate management of trauma-induced infections is associated with the development of chronic osteomyelitis (CO), a complication that causes morbidity and results in reduced quality of life [8].

The diagnosis of osteomyelitis is established through a comprehensive assessment, including clinical history and physical examination, biochemical investigations of inflammatory and blood markers, and imaging studies [7]. The effective diagnosis of osteomyelitis is primarily achieved through the integration of simple radiography with a congruent medical history and a thorough physical examination. Scintigraphy and magnetic resonance imaging (MRI) are beneficial in difficult-to-diagnose conditions; MRI remains the gold standard for distinguishing collections and adjacent edema. Although radiographic findings exhibit classical features, they lack specificity. Most skeletal pathologies exhibit common observable characteristics such as cortical thickening or thinning, rarefaction of bone trabeculae due to tissue resorption, localized soft tissue edema, and alterations in adjacent joints. Furthermore, accurate differentiation from neoplastic lesions is imperative considering the potential consequences in the event of a diagnostic misjudgment [9]. Radiographic findings on plain radiographs vary depending on the disease phase. During the acute phase, notable findings include deep soft-tissue swelling, periosteal reaction, cortical irregularity, and varying degrees of demineralization. Conversely, the chronic phase manifests distinct alterations in the bone structure, such as irregular tissue with evidence of destruction or bone lysis, cortical thickening or thinning, sclerotic bone interspersed with radiolucencies, elevated periosteum, presence of sequestrum, and the occurrence of draining sinuses [10]. Single or multiple radiolucent abscesses, cortical sequestration, and new bone apposition are the characteristic findings in CO [11].

This study aimed to assess and compare the proficiency of orthopedic surgeons and radiologists in identifying radiographic signs using simple radiographs. The emphasis on radiography as a diagnostic tool has declined owing to the recent technological advancements in the field. Therefore, this study aimed to underscore the continued importance and utility of plain radiography as a rapid, cost-effective, and readily accessible examination in the diagnostic process.

## Methods

This was a correlation study using plain radiographs of patients diagnosed with CO. Between November 2018 and November 2020, data were collected from a reference center for osteomyelitis at a university hospital in southern Brazil. This study was approved by the Ethics Committee of the UFPR (Curitiba, Brazil) (CAAE:81,162,817.2.0000.0020). The requirement for informed consent was waived by the ethics committee and the Institutional Review Board that approved this study. This study was approved by the local ethics committee, and informed consent was waived owing to the non-interventional design of the study.

The study population comprised 45 men and 15 women (mean age, 45.1 years; range: 17–79 years). This cross-sectional study included 60 patients who were referred to our hospital with a clinical suspicion of active osteomyelitis in the bone that was previously affected by fracture, infection, and surgery. Patients with joint prostheses, pressure ulcers, or diabetic feet were excluded. Radiographs were available for all the patients. The final diagnosis of active osteomyelitis was confirmed based on the surgical findings and biopsy results (pathological or microbiological study).

**Fig. 1:**
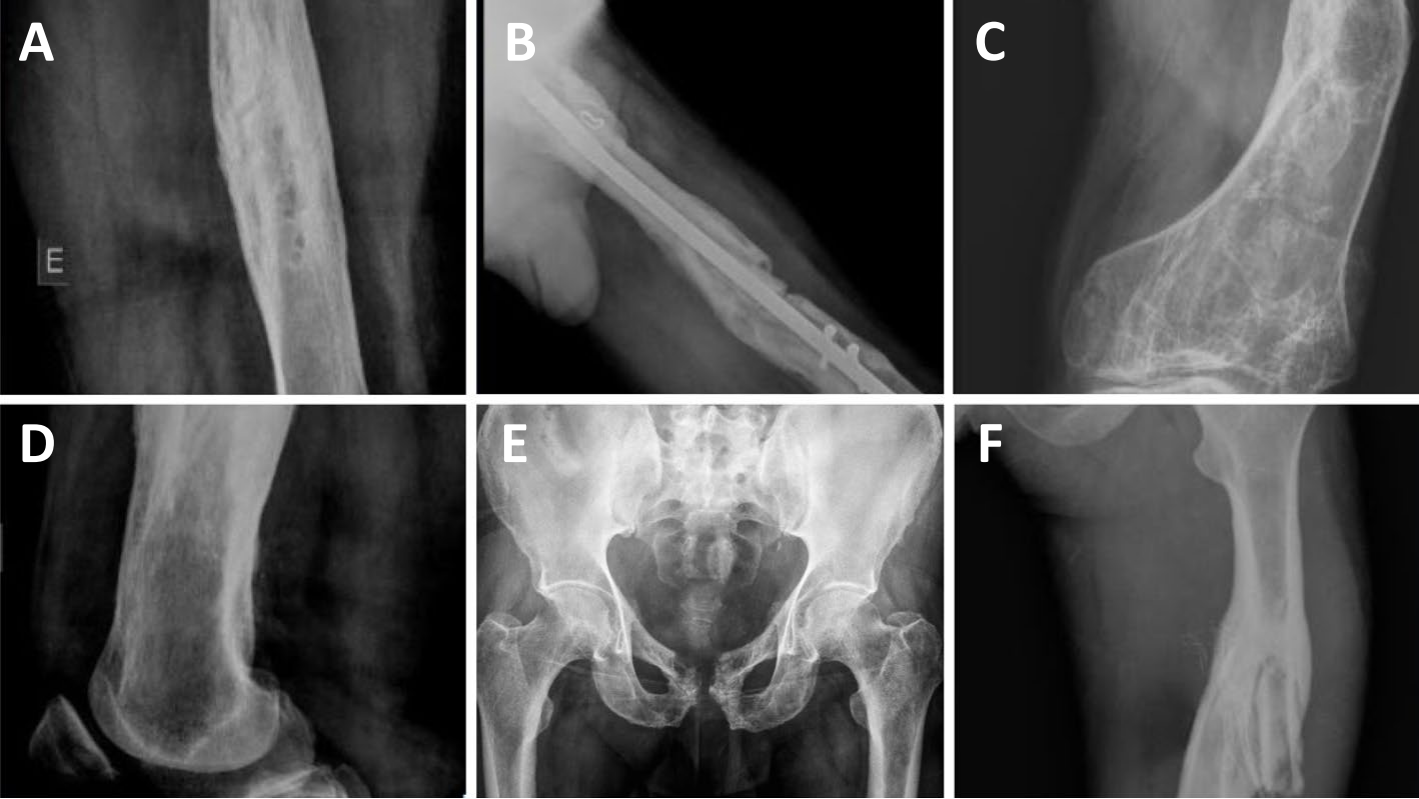
Radiographic findings evaluated in patients with chronic osteomyelitis. **A** bone sclerosis, **B**) soft-tissue swelling, **C**) cortical thinning (with erosion), **D**) cortical thickening, **E**) bone destruction, **F**) sequestrum

All radiographs were interpreted prospectively by three orthopedic surgeons and three radiologists with relevant knowledge about CO diagnosis. No additional data was provided. Radiographs from patients were obtained at the time of initial admission, before any treatment or surgical intervention. We recorded the presence or absence of the following six findings: bone destruction (including erosion and radiolucencies around the implants), bone sclerosis, cortical thinning (with erosion), cortical thickening, sequestrum, and soft-tissue swelling [9] (Fig. 1).

The demographic and clinical characteristics, comorbidities, and microbiological parameters were evaluated. Other findings included the CO site, presence of a fistula, disease duration, and presence of pseudoarthrosis.

Continuous variables are expressed as averages with ranges (normal distribution after the Kolmogorov normality test). Categorical variables are expressed as absolute frequencies and proportions. The interobserver agreement between the radiographs was evaluated using the scores given by each physician and expressed as median values and interquartile ranges (IQR) for each radiographic finding.

## Results

**Fig. 2:**
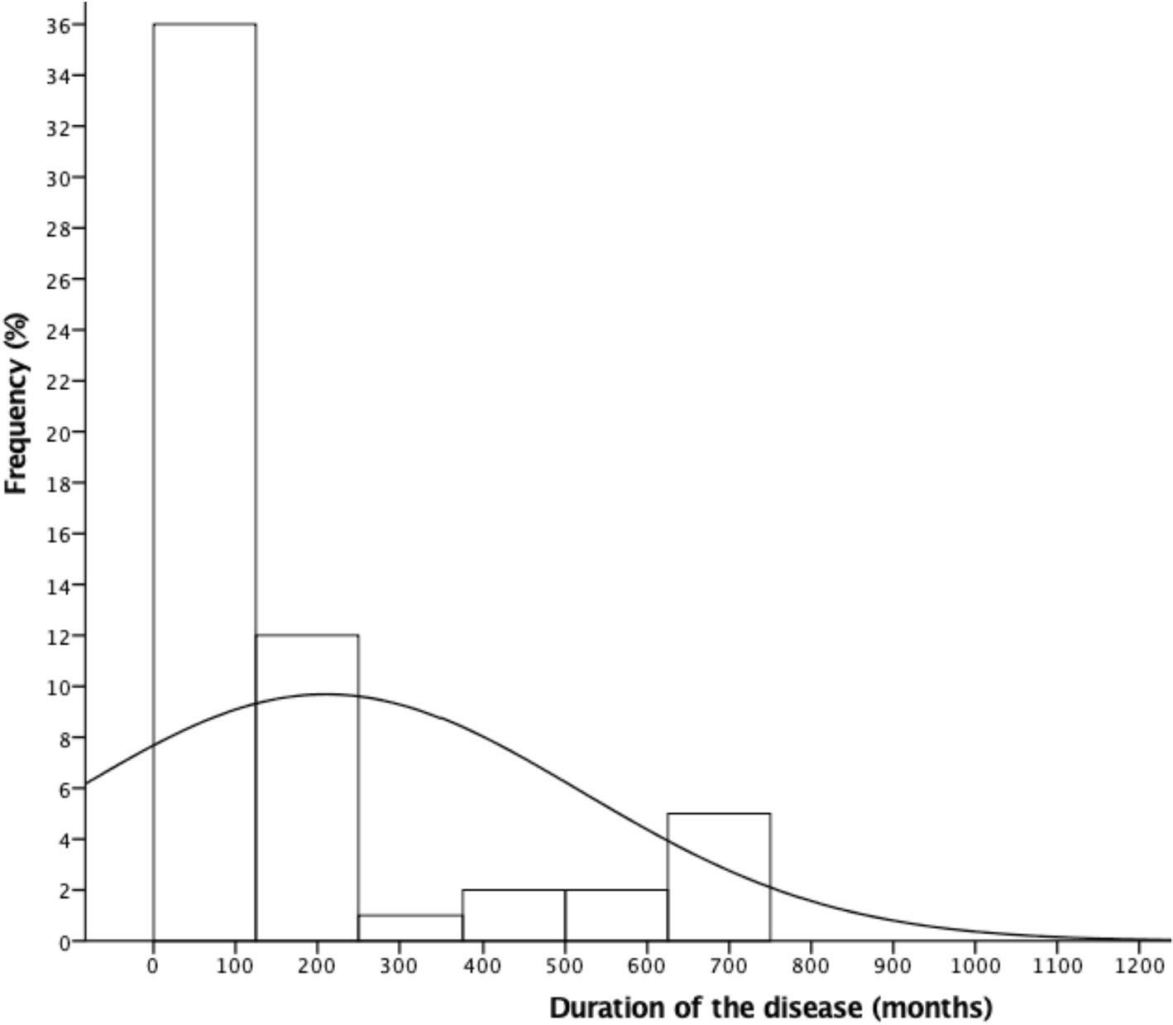
Histogram of the duration of the chronic osteomyelitis symptoms

**Fig. 3:**
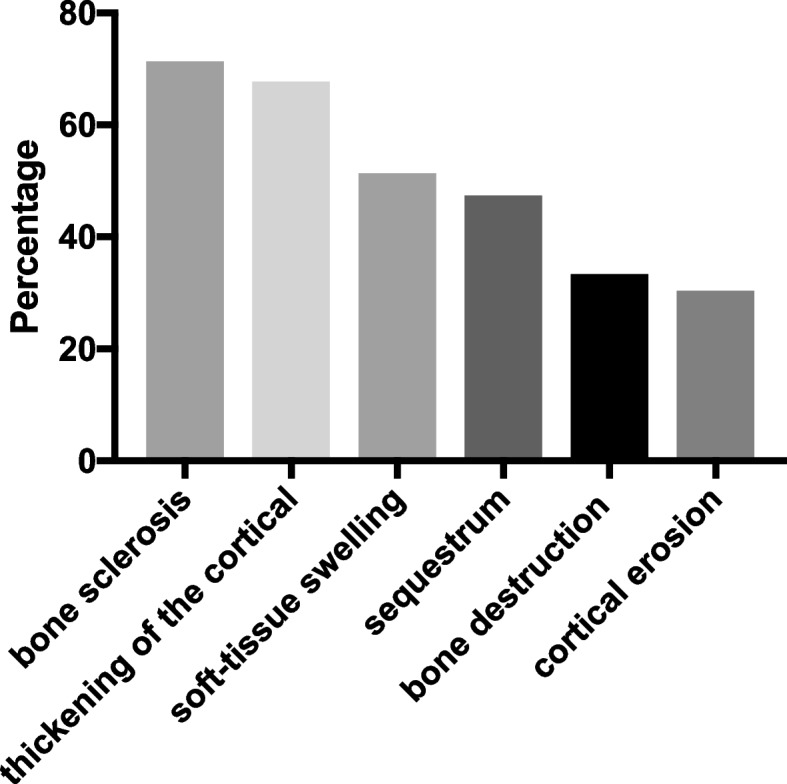
Frequency of radiograph alteration in 60 patients with chronic osteomyelitis

In this cross-sectional study, most patients were men (75%), with an average age of 45.1 years. CO most frequently occurs in the tibia (58%) and is secondary to trauma. Hematogenous etiology of CO represented 23%. The average duration of disease before admission to the reference center was 210 months (range: 5–1452 months) (Fig. 2). Arterial hypertension was the most common comorbidity (22%). The other clinical and laboratory characteristics are summarized in Table 1. Bone sclerosis (71.3%) and cortical thickening (67.7%) were the most common radiographic findings, followed by soft tissue swelling (51.3%), sequestrum formation (47.3%), bone destruction (33.3%), and cortical erosion (30.3%) (Fig. 3). Regarding the radiographic characteristics with and without implants, the assessment was limited to bone analysis, and we confirmed that there was no difference. There was no radiographic difference based on the identified pathogen in cultures.

**Table 1:** Clinical and laboratory characteristics of 60 patients with chronic osteomyelitis

**Variable**	***n* = 60**
Average age (years) with range	45.1 (17–79)
Male gender	45 (75%)
Chronic osteomyelitis	
Single site/multiply sites	57/3 (97%/3%)
Single site	
Tibia	35 (58%)
Femur	21 (35%)
Other	4 (7%)
Trauma/hematogenous	46/14 (77%/23%)
Average duration of infection (months)	210 (5—1452)
Rate of sinus	52 (86%)
Rate of bone defect or nonunion	5 (8%)
Positive rate of culture	26 (43%)
Most common bacteria	S. aureus (6/26)
White blood cells (thousands/microL)	7290 ± 2455
C-Reactive Protein (mg/L)	4.4 ± 8.9
Erythrocyte sedimentation rate (mm/hr)	28.5 ± 35.83
HIV	0 (0%)
Diabetes	5 (8%)
Chronic renal failure	1 (2%)
Heart failure	1 (2%)
Chronic pulmonary obstructive disease	1 (2%)
Syatemic arterial hypertension	13 (22%)
Neoplasm	0 (0%)
Reumathological disease	1 (2%)
Cirrhosis	0 (0%)

**Fig. 4:**
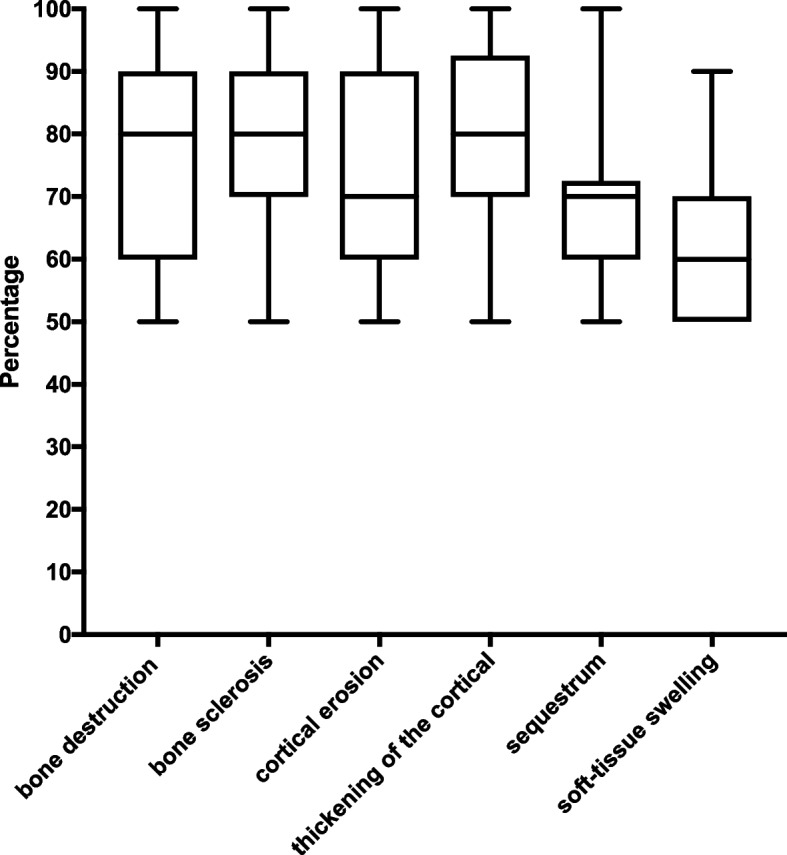
Percentage of agreement among specialists (orthopedists and radiologists) of presence or absence of radiograph alteration in chronic osteomyelitis

Regarding the agreement of the presence or absence of lesions, we found that the mean agreement was 74.2%, showing a marked rate of disagreement (25.8%) among all radiographic findings. The interobserver agreement for the type of radiographic finding was the highest for bone sclerosis (80.7%) and cortical thickening (80.3%). The presence or absence of soft tissue edema, a prominent radiographic finding among the other findings, showed the greatest disagreement (Fig. 4).

## Discussion

The diagnostic assessment of CO encompasses a spectrum of imaging modalities ranging from plain radiography to nuclear imaging techniques. Plain radiographs provide an excellent means of bone evaluation and are characterized by widespread availability and cost-effectiveness [12]. Therefore, simple radiography is considered the primary imaging modality in the initial evaluation of suspected musculoskeletal infections and is beneficial for the differential diagnosis of bone lesions and osteomyelitis [13]. Plain radiography remains the most suitable initial examination, frequently providing the essential information required to guide the management of osteomyelitis [10].

The radiological manifestations of osteomyelitis vary according to disease progression through the acute, subacute, and chronic phases. The sensitivity and specificity of plain radiography are 43%–75% and 75%–83%, respectively [14]. The sensitivity of plain radiography during the acute phase is less than 5%, with approximately 30% at one week, which further increased to 90% after 3–4 weeks. Therefore, simple radiography is effective in monitoring CO [15].

Certain diseases can induce alterations in bone trabecular and cortical architecture, which are typically observed on plain radiographs when there is a loss of at least 30% of bone density [11, 14], and lytic lesions become apparent when 75% of the bone matrix is destroyed [10]. Stress fractures, osteoid osteomas, Ewing's sarcoma, eosinophilic granuloma, and periostitis can mimic the symptoms of both acute and chronic osteomyelitis. Additionally, specific infectious bone conditions, such as Brodie’s abscess, may manifest as subacute osteomyelitis, particularly in the metaphyses near the growth plate in the immature skeleton. The characteristic radiographic features of osteomyelitis include associated soft tissue swelling, a variable degree of periosteal reaction, and a central area of radiolucency surrounded by a thick rim of reactive bone sclerosis [15]. Sclerosing osteomyelitis of Garré is a chronic disease that affects the mandible and long bones, presenting with diffuse sclerosis and bone thickening [15].

In the context of chronic bone infections, several specific terms merit definition. A “sequestrum” refers to a segment of deceased bone that has become detached from the viable bone, typically existing independently, and is surrounded by granulation tissue. Conversely, an “involucrum” denotes the formation of new bone on the surface of an existing dead bone or sequestrum. An opening within an involucrum is termed a "cloaca," which serves as the origin of a sinus leading from the sequestrum to the exterior of the bone [12]. In our study, we identified six most common radiographic findings in patients with CO. Bone sclerosis (71.3%) and cortical thickening (67.7%) were the two most common radiographic findings, followed by soft-tissue swelling (51.3%), sequestrum (47.3%), bone destruction (33.3%), and cortical erosion (30.3%) (Fig. 2). We considered bone sclerosis and cortical thickening as two specific bone alterations associated with CO. Bone sclerosis is a considerable alteration in the diagnosis of CO; however, this finding may be present in various congenital, autoimmune, and other rare diseases [16]. Similarly, cortical thickening, the second-most prevalent radiographic sign of CO, is subject to interpretation. Cortical thickening may also be manifested in bone metabolism disorders, medication-induced changes, or alterations associated with vitamin-related deficiencies [17]. Therefore, radiographic changes alone are insufficient to diagnose osteomyelitis, emphasizing the importance of the clinical presentation, medical history, and physical examination.

Tumeh et al. [9] advocated that the presence of a sequestrum is highly specific for diagnosing active osteomyelitis. Although we acknowledge Tumeh’s perspective, our observations diverge, as the presence of a sequestrum in many cases does not unequivocally signify ongoing activity at the time of evaluation. In contrast, we speculate that the presence of a sequestrum, particularly when coupled with local acute symptoms such as pain, edema, and erythema, serves as compelling evidence of current activity, warranting acute surgical intervention.

Soft tissue swelling, a primary indicator of local bone infection, is one of the earliest signs observed on plain radiography. Despite its lack of specificity for indicating bone infection, it was significant in our study, ranking as the third most frequently observed sign. Conversely, other findings, such as the sequestrum, bone destruction, and cortical erosion, underscore the severity of the infection. The presence of a sequestrum suggests chronicity, whereas bone destruction and cortical erosion indicate the extent of compromised bone architecture and diminished mechanical resistance.

The most noteworthy discrepancy in the presence or absence of soft tissue edema emerged as the most prominent radiographic finding in our study. We presume that this particular finding is inherently subjective and aligns with existing literature. However, consensus among the specialists was observed in the evaluation of bone destruction and bone sclerosis, underscoring the importance and severity of these observed changes.

Our study had several limitations. First, the inclusion of only six specialists; however, we contend that the addition of more physicians may not significantly alter the percentages unless a reduction in the standard deviation occurs. Additionally, we excluded osteomyelitis occurring in less common sites, such as the shoulders and feet, owing to its rarity and increased interpretational complexity. The sample size could potentially be higher; however, the stringent inclusion criteria coupled with the capacity constraints of our specialized service dictated the sample size in our study. In addition, individuals with severe and chronic bone infections from across the state were referred to the reference center for osteomyelitis treatment, which contributed to an inherent bias toward the inclusion of patient cohort with prolonged diseases and interrupted treatment.

In summary, our study underscores the importance of plain radiographs for both therapeutic guidance and monitoring the progression of CO. The primary radiographic alterations identified in this study were bone sclerosis and cortical thickening. Despite the variability in agreement among the different alterations, significant differences were not observed. Nonetheless, more advanced imaging modalities, including computed tomography, magnetic resonance imaging, and bone scintigraphy, are deemed beneficial and recommended for the comprehensive evaluation and judicious treatment of CO.

## Data Availability

The datasets used and/or analyzed during the current study are available from the corresponding author on reasonable request.
